# Spatial Retrieval of Broadband Dielectric Spectra

**DOI:** 10.3390/s18092780

**Published:** 2018-08-23

**Authors:** Jan Bumberger, Juliane Mai, Felix Schmidt, Peter Lünenschloß, Norman Wagner, Hannes Töpfer

**Affiliations:** 1 Department Monitoring and Exploration Technologies, Helmholtz Centre for Environmental Research—UFZ, Permoserstrasse 15, 04318 Leipzig, Germany; felix.schmidt@ufz.de (F.S.); peter.luenenschloss@ufz.de (P.L.); 2 Department of Computational Hydrosystems, Helmholtz Centre for Environmental Research—UFZ, Permoserstrasse 15, 04318 Leipzig, Germany; juliane.mai@uwaterloo.ca; 3 Institute of Material Research and Testing—MFPA at the Bauhaus-University Weimar, Coudraystrasse 9, 99423 Weimar, Germany; norman.wagner@mfpa.de; 4 Department of Advanced Electromagnetics, Technische Universität Ilmenau, Helmholtzplatz 2, 98693 Ilmenau, Germany; hannes.toepfer@tu-ilmenau.de

**Keywords:** electromagnetic scattering inverse problems, microwave propagation, dielectric materials, dielectric measurements, soil measurements, modeling

## Abstract

A broadband soil dielectric spectra retrieval approach (1 MHz–2 GHz) has been implemented for a layered half space. The inversion kernel consists of a two-port transmission line forward model in the frequency domain and a constitutive material equation based on a power law soil mixture rule (Complex Refractive Index Model—CRIM). The spatially-distributed retrieval of broadband dielectric spectra was achieved with a global optimization approach based on a Shuffled Complex Evolution (SCE) algorithm using the full set of the scattering parameters. For each layer, the broadband dielectric spectra were retrieved with the corresponding parameters thickness, porosity, water saturation and electrical conductivity of the aqueous pore solution. For the validation of the approach, a coaxial transmission line cell measured with a network analyzer was used. The possibilities and limitations of the inverse parameter estimation were numerically analyzed in four scenarios. Expected and retrieved layer thicknesses, soil properties and broadband dielectric spectra in each scenario were in reasonable agreement. Hence, the model is suitable for an estimation of in-homogeneous material parameter distributions. Moreover, the proposed frequency domain approach allows an automatic adaptation of layer number and thickness or regular grids in time and/or space.

## 1. Introduction

**Figure 1:**
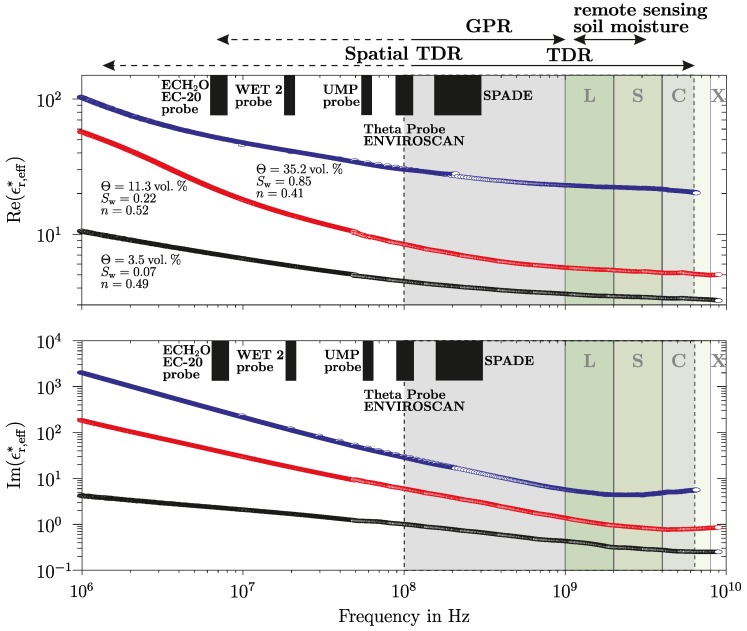
Complex relative effective permittivity $\epsilon_{r,\mathrm{eff}}^{*}$ of a silty clay loam with different volumetric water contents θ or water saturations $S_{w}$ and porosities *n* (figure according to [25]). L, S, C and X are the names of frequency bands and the black blocks depict the narrow frequency ranges of typical soil moisture probes.

Since the late 1970s, radar remote sensing methods were routinely used for the observation of the land surface with satellites or airborne-based radar [1,2,3,4,5,6,7] and the near and sub-surface with Ground-Penetrating Radar (GPR, [8,9,10,11,12,13]). The High Frequency Electromagnetic (HF-EM) properties of the soil and rock formations determine the Electromagnetic (EM) response of the radar signals [14,15,16,17,18]. A quantitative temporal and spatial retrieval of HF-EM material properties requires a validation against in situ measurements [19]. For this purpose, minimally-invasive ground-based EM time or frequency domain sensor techniques are used [11,20,21,22,23,24]. For the successful application and combination of these techniques, which operate in different frequency bands, the knowledge of the broadband frequency and temperature-dependent HF-EM properties are needed (see Figure 1).

In general, an EM signal with a bandwidth from 1 MHz–10 GHz contains valuable information of a measured porous material due to the contribution to the dielectric relaxation behavior by the [6,15,18,26,27]:

(i) Bulk HF-EM properties of the volume fractions (solid particles, air, aqueous pore solution),
(ii) Geometrical properties of the bulk phases (particle shape, pore size distribution),
(iii) Mobility of charges in the pore network (extra and intra-aggregate porosity) from nm–μm and
(iv) Interactions between the aqueous pore solution and mineral phases due to interface effects.

This opens the possibility to estimate physicochemical parameters from a broadband dielectric spectrum of the material besides volumetric water content such as porosity, texture, mineralogy and hydraulic properties with broadband HF-EM measurement techniques. However, there is a lack of studies or approaches for the spatial estimation of broadband dielectric spectra.

A useful method for the retrieval of the spatial distribution of HF-EM material properties is called spatial time domain reflectometry [28,29,30,31,32,33,34,35]. As measurements are achieved with a Time Domain Reflectometry (TDR) instrument, available modeling and inversion algorithms operate primarily in the Time Domain (TD) with direct full waveform inversion of the TDR signal [36], numerical finite difference forward modeling and optimization in the time domain [30,31] or transmission line modeling in the Frequency Domain (FD) in association with optimization in the time domain [29,32,33].

The underlying modeling concept is based on an equivalent circuit formulation of the telegrapher’s equation for the propagation of a plane EM wave in the Transverse EM mode (TEM) without considering the frequency dependence [30] or considering the frequency or an equivalent time dependence based on a Debye relaxation function in the forward problem on a regular grid [29,30,32,33,37]. Moreover, full wave numerical approaches for the 1D, 2D or 3D HF-EM calculation of EM response related to sensor developments provide alternative approaches [23]. However, due to non-Debye dispersion and absorption in moist porous materials, the constitutive material equations resulting in a fractional order of the differential operator, the resultant governing partial differential equations for EM wave propagation become fractional [38,39,40,41,42,43]. This issue is not considered in available TD solvers. In contrast, FD solvers overcome this inconsistency by solving the set of equations at a specific frequency, which allows the incorporation of the appropriate material properties without any restriction [44,45].

This allows the numerical determination of the HF-EM transfer function of complex structures in terms of scattering parameters with high precision in comparison to measurements, which is shown for combined numerical and experimental investigations in the case of homogeneous samples measured with the broadband frequency domain transmission line technique in [25,46,47]. However, due to the full 3D wavelength-based adaptive mesh refinement, at least small measurement configurations require high computational costs in terms of memory and time, which currently are not suitable for forward models with spatial dimensions in the meter scale. Against this background, 1D transmission line modeling in the frequency domain considering non-Debye-type relaxation offers an appropriate alternative [47,48,49,50,51].

In this study, an FD transmission line modeling for the determination of the scattering matrix of a two-port coaxial transmission line with three segments is presented. We considered the broadband dielectric spectrum in terms of the soil based on a theoretical power law mixture model. For the spatially-resolved retrieval of the broadband dielectric spectra, an inverse modeling scheme based on a Shuffled Complex Evolution (SCE) algorithm was applied.

## 2. Materials and Methods

### 2.1. Transmission Line Forward Model

The propagation of an EM wave along a transmission line was characterized by a scattering matrix $S=(S_{ij})$. The four complex scattering parameters $S_{ij}$ with i,j∈{1;2} were modeled based on the frequency-temperature-pressure-dependent complex effective permittivity $\epsilon_{\mathrm{eff}}$ of the material and layer thickness following [52] using EM propagation matrices [53].

**Figure 2:**
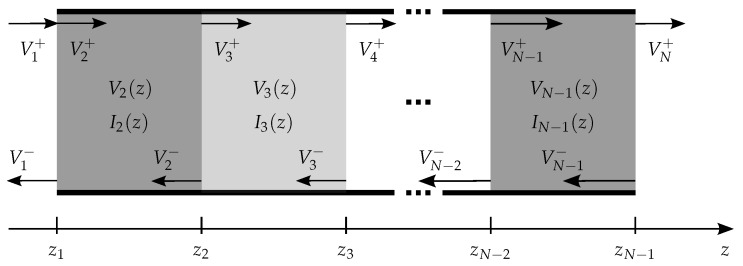
Schematic representation of the layered transmission line.

In order to describe the 1D propagation of a transverse EM wave perpendicular to parallel layers of thickness $d_{k}$, with $\left\{N;k\in N^{+}|k\leq N-1\right\}$, the transmission line was divided into *N* sections (Figure 2). As known from transmission line theory, for every homogeneous section *k*, which extends between the points $z_{k-1}$ and $z_{k}$ with $d_{k}=z_{k}-z_{k-1}$, the complex phasors of voltage $V_{k}$ and current $I_{k}$ in section *k* are given by:

(1) $$\begin{array}{c} V_{k}\left(z\right)=V_{k}^{+}e^{-\gamma_{k}\left(z-z_{k-1}\right)}+V_{k}^{-}e^{-\gamma_{k}\left(z_{k}-z\right)}, \end{array}$$

(2) $$\begin{array}{c} I_{k}\left(z\right)=V_{k}^{+}Z_{k}^{-1}e^{-\gamma_{k}\left(z-z_{k-1}\right)}-V_{k}^{-}Z_{k}^{-1}e^{-\gamma_{k}\left(z_{k}-z\right)}. \end{array}$$

Here, *z* is the distance along the transmission line and the complex phasors and $V_{k}^{+}$ and $V_{k}^{-}$ express the forward and backward traveling voltage waves coming from adjacent sections. The material properties of the *k*-th line section were considered by the permittivity dependency of the propagation factor $\gamma_{k}$ and the characteristic impedance $Z_{k}$. For a coaxial cell, they are given by:

(3) $$\gamma_{k}=\frac{i\omega }{c_{0}}\sqrt{\epsilon_{r,k}^{*}}\;\wedge \;Z_{k}=\frac{Z_{0}}{\sqrt{\epsilon_{r,k}^{*}}}.$$

Herein, $\epsilon_{r,k}^{*}=\epsilon_{r,k}^{\prime }-i\epsilon_{r,k}^{\prime \prime }$ is the complex relative dielectric permittivity of the *k*-th layer; where the real part of $\epsilon_{r,k}^{*}$ represents capacitive effects caused by polarization and the imaginary part refers to appropriate dielectric and electrical losses in the material. Furthermore, ω represents the angular frequency; $c_{0}$ is the velocity of light in free space; and $Z_{0}$ is the reference impedance of the empty transmission. In Equation (3), the square root has 2 solutions. For further calculations, the solutions with the negative imaginary part were used, leading to a positive constant attenuation factor, respectively, in $\gamma_{k}$. Furthermore, $V_{k}^{+}$ and $V_{k}^{-}$ represent the voltages at the *k*-th interface between *N* layers and were combined into one vector:

(4) $$\begin{array}{c} V_{k}=\left[\begin{array}{c} V_{k}^{+}\\ V_{k}^{-} \end{array}\right]. \end{array}$$

In [52], an expression is given that relates the waves at the first intersection $V_{1}$ with the ones at the end $V_{N}$:

(5) $$V_{1}=\underset{:=A}{\underset{︸}{{\left(M_{1}^{L}\right)}^{-1}\left[\prod_{k=2}^{N-1}M_{k}^{R}{\left(M_{k}^{L}\right)}^{-1}\right]M_{N}^{R}}}V_{N}.$$

The matrices $M_{k}^{R}$ and $M_{k}^{L}$ are given as follows:

(6) $$\begin{array}{c} M_{k}^{R}=\left[\begin{array}{cc} e^{-\gamma_{k}d_{k}} & 1\\ Z_{k}^{-1}e^{-\gamma_{k}d_{k}} & -Z_{k}^{-1} \end{array}\right], \end{array}$$

(7) $$\begin{array}{c} M_{k}^{L}=\left[\begin{array}{cc} 1 & e^{-\gamma_{k}d_{k}}\\ Z_{k}^{-1} & -Z_{k}^{-1}e^{-\gamma_{k}d_{k}} \end{array}\right]. \end{array}$$

With the scattering parameter:

(8) $$S_{11}=\frac{V_{1}^{-}}{V_{1}^{+}}\;\wedge \;S_{21}=\frac{V_{N}^{+}}{V_{1}^{+}},$$

Equation (5) is written as:

(9) $$\begin{array}{c} \left[\begin{array}{c} 1\\ S_{11} \end{array}\right]=A\left[\begin{array}{c} S_{21}\\ 0 \end{array}\right]. \end{array}$$

Here, the forward wave $V_{1}^{+}$ at port 1 was set to 1. This is possible because network analyzers do not measure absolute values, but quotients, e.g., the reflected to incident power wave. This system of equations for the *S*-parameters gives:

(10) $$S_{11}=\frac{A_{21}}{A_{11}}\;\wedge \;S_{21}=\frac{1}{A_{11}}.$$

Assuming a perfectly symmetric and homogeneous line, S is symmetric. The scattering parameters $S_{22}$ and $S_{12}$, which refer to the measurement from the other side of the inhomogeneous transmission line, were modeled in analogy by inverting the layer sequence. The new layer parameters depicted with inv were calculated as follows:

(11) $$\gamma_{k}^{\mathrm{inv}}=\gamma_{N-k}\;\wedge \;Z_{k}^{\mathrm{inv}}=Z_{N-k}\;\wedge \;d_{k}^{\mathrm{inv}}=d_{N-k}.$$

### 2.2. Broadband Constitutive EM Model

In general, the complex relative effective dielectric permittivity $\epsilon_{r,\mathrm{eff}}^{*}$ of a multi-phase material containing an aqueous solution depends on temperature, pressure and frequency. The broadband electrical dispersion and absorption of a moist soil had been approximated with the theoretical power law model [25,54] using an exponent equal to 1/2 (Complex Refraction Index Model (CRIM)).

(12) $$\sqrt{\epsilon_{r,\mathrm{eff}}^{*}\left(S_{W},\omega ,n\right)}=S_{W}\;n\;\sqrt{\epsilon_{r,W}^{*}\left(\omega \right)}+\left(1-n\right)\sqrt{\epsilon_{r,G}^{*}}+n\left(1-S_{W}\right).$$

Here, $\epsilon_{r,W}^{*}$ is the complex relative permittivity of the aqueous pore solution and $\epsilon_{r,G}^{*}$ the complex relative permittivity of the mineral matrix, e.g., solid particles. $S_{W}$ is the saturation of water in the pore room, and *n* is the porosity. This formula is a weighted sum of the properties related to the travel time of the EM wave through the three materials: aqueous pore solution, the solid particles (e.g., quartz, feldspar, mica, kaolinite, vermiculite [46]) and air. It is only valid if the mixture is homogeneous and isotropic at the sample scale, which is itself related to the applied wavelength.

The pore water was assumed to be the only frequency-dependent material in this mixture in the considered temperature and pressure range. The dielectric relaxation behavior $\epsilon_{r,D}^{*}\left(\omega \right)$ was modeled by a Debye-type function (see [26,55,56]) that is valid for water in the studied frequency, temperature and pressure range [56,57,58]:

(13) $$\epsilon_{r,D}^{*}\left(\omega \right)=\epsilon_{\infty }+\frac{\epsilon_{s}-\epsilon_{\infty }}{1+i\omega \tau }.$$

Here, $\epsilon_{\infty }$ is the high-frequency permittivity, $\epsilon_{s}$ the static permittivity and τ the dielectric relaxation time, with $\tau ={\left(2\pi f_{\mathrm{rel}}\right)}^{-1}$, where $f_{\mathrm{rel}}$ is the relaxation frequency. Furthermore, pore water results in a considerable direct current electrical conductivity $\sigma_{W}$, which contributes to the losses [26]:

(14) $$\epsilon_{r,W}^{*}=\epsilon_{r,D}^{*}\left(\omega \right)-i\frac{\sigma_{W}}{\omega \epsilon_{0}}=\epsilon_{r,D}^{\prime }-i\left(\epsilon_{r,D}^{\prime \prime }+\frac{\sigma_{W}}{\omega \epsilon_{0}}\right).$$

The broadband three-phase CRIM model used includes water relaxation, interfacial relaxation, as well as losses due to direct-current conductivity. From a practical point of view, the supposed model is sufficient for most soils and rocks in the considered frequency range from 1 MHz–10 GHz [25,26,44,47].

### 2.3. Retrieval Approach

We used an optimization algorithm based on an analytical forward model of the scattering parameters $S_{ij}^{\mathrm{model}}$ to fit the measurement $S_{ij}^{\mathrm{meas}}$. This was done by summing up the square errors for all four $S_{ij}$-parameters over all the frequency points $\omega_{k}$ (*Q* is the number of frequency points) without a weight function. In this study, we employed the objective function:

(15) $$F\left(p\right)=\sum_{i,j=1}^{2}\sum_{k=1}^{Q}{∥S_{ij}^{\mathrm{model}}\left(p,\omega_{k}\right)-S_{ij}^{\mathrm{meas}}\left(\omega_{k}\right)∥}^{2}\;\Rightarrow \;\mathrm{Min}$$

to minimize the distance between the measured and modeled scattering parameters $S_{ij}$. The distance was calculated as the sum of all squared differences between the measured and observed scattering values $S_{ij}$. Here, *p* is the parameter set and $∥\;\cdot \;∥$ the complex absolute value function. The optimal parameter sets *p* were inverted using the shuffled complex evolution algorithm (see the following Section 2.3.1). Subsequently, the uncertainty of this optimal parameter set was determined (see the following Section 2.3.2). The two methods employed are described in the following.

#### 2.3.1. Inverse Parameter Estimation

For the variation of the parameters, the Shuffled Complex Evolution (SCE) algorithm introduced by Duan et al. [59] with parameter adjustments of Behrangi et al. [60] was used. The strategy of this algorithm is to form so-called complexes (e.g., triangles in 2D) out of M+1 parameter sets *p*, where *M* is the number of model parameters. Each vertex of the complex does not only represent one of the M+1 parameter sets, but also the model’s fitness ability to match the observed data when it is supplied with the according parameter set. This fitness ability is usually referred to as being the objective function value of an objective to be minimized. The fitness ability with the worst fitness ability or largest objective function value is subsequently perturbed in order to find a better substitute parameter set. This strategy is repeated until the area of the complex, i.e., the agreement of the parameter sets, is smaller than a threshold. In order to avoid that the search gets stuck in a local optimum, a number of *C* complexes are acting in parallel. After a certain number of iterations, the C×(M+1) vertexes are shuffled and newly assigned to *C* complexes. The algorithm converges when the volume of all complexes is lower than a threshold, which means that all C×(M+1) vertexes are in close proximity to each other.

The SCE algorithm used here was configured with two complexes, each consisting of (2M+1) ensemble members with *M*. In each iteration, M+1 parameters were randomly selected, and the vertex with the worst fitness ability was perturbed. The perturbation itself can be interpreted as geometric transformations of these complexes. Possible transformations are reflection, contraction and expansion. All these transformations perturb only the vertex with the worst fitness. Two algorithmic constants have to be set for these geometric transformations, i.e., the reflection step length ξ and the contraction step length ζ. We chose the values ξ=0.8 and ζ=0.45 used by Behrangi et al. [6]. SCE seems to have an order of about $O\left(M^{2}\right)$. In our case, it required between $5\times 10^{4}$ and $2.5\times 10^{5}$ model evaluations to find the optimal set of the *M* parameters. The selection of SCE was based on its widespread usage in hydrological studies and according to a preliminary experiment where the SCE outperformed other algorithms like simulated annealing [61] and the dynamically-dimensioned search algorithm [62] in optimizing more than 80 analytical test functions with *M* ranging from 2–30.

#### 2.3.2. Uncertainty Estimation

Uncertainty analysis methods determine the likely range of an optimized parameter. The impossibility to specify one single, best parameter set after optimization originates from the fact that erroneous data are used to constrain the parameters, and these errors propagate into the parametric uncertainty. This means that the data points are more correctly probability distributions with a certain mean, usually the measured data point, and a given spread, usually the known data error. These data probability distributions directly translate into parameter probability distributions. Uncertainty estimation methods such as the Monte Carlo Markov Chain (MCMC) methods [63] are used to estimate these parameter distributions. Therefore, parameter sets are sampled, and the respective simulation is performed. Subsequently, the likelihood of the parameter set was estimated through the comparison of the modeled output with the known data distributions. Parameter sets with low likelihoods are rejected, while the others are retained and used as new starting points for sampling the next candidate parameter set. The MCMC thus explores the parameter domain and collects parameter sets of comparable skill. Finally, all the retained parameter sets are used to build the requested parameter distributions.

The algorithm used here started with a burn-in phase tuning the step sizes per parameter according to the method described by [64]. The range of the acceptance ratio was adjusted from [0.25, 0.35] to [0.23, 0.44] as proposed by [63]. An additional criterion for the acceptance ratios was introduced to assure the convergence of these values. Therefore, it was checked if the variance of the ratios of the last ten iterations was below a certain threshold. This criterion was tested for each parameter independently. The burn-in phase of the MCMC was terminated if all ratios were in Gelman’s range [63] and the variance was small enough. Then, the step sizes were fixed and used to sample new candidate parameter sets in the proper MCMC sampling. In total, L=5 parallel MCMC chains were used. During every 1000×LM model evaluations, it was checked whether all parameters met the Gelman–Rubin criterion [63]:

(16) $$\mathrm{GR}=\sqrt{\frac{(l-1)/n\;\cdot \;W+1/l\;\cdot \;B}{W}}<1.1$$

where *W* is the within-chain variance, *B* is the between-chain variance and *l* is the number of parameter sets used to calculate this statistic. Always, only every tenth parameter set of the second half of each chain was taken to guarantee independence of the parameter sets. To allow the algorithm to move away from the starting point, only the second half of all sampled parameter sets of each chain was considered. Thus, *l* was always 0.05×m with *m* being the total number of parameter sets per chain. The MCMC was terminated when all parameters satisfied the Gelman–Rubin criterion. Otherwise, additional 1000×LM parameter sets were sampled and evaluated. After convergence of the algorithm, every tenth parameter set of the second half of all L=5 chains was used to determine the standard deviation per parameter that was later referred to as the uncertainty of the respective parameter. In this study, a total of 5×l parameter sets were evaluated, which represents between $8\times 10^{4}$ and $8\times 10^{5}$ parameter sets. The number of sets depends on the parametrization model used.

### 2.4. Experiments

In order to demonstrate the performance of the developed forward modeling and inversion method, two validations are demonstrated in this section. The first was a laboratory experiment based on Vector Network Analyzer (VNA) measurement of a coaxial cell partly filled with nearly non-dispersive Polytetrafluorethylene (PTFE). It was designed to test the multilayer frequency domain inversion approach in general. The second numerical experiment used synthetic data with a dispersive electrically-lossy mixture assuming four relevant hydrogeological scenarios. Here, the broadband dielectric spectra including the corresponding parameter porosity, water saturation, complex permittivity of the solid material and electrical conductivity of the aqueous pore solution were reconstructed simultaneously for each layer to analyze the method with environmental materials analogous to soil.

#### 2.4.1. Experiment 1: Coaxial Cell Measurements with Constant Permittivity

The frequency domain measurements were carried out with the VNA (E5071C ENA Series, Agilent Technologies, Santa Clara, CA, USA). The frequency bandwidth was set from 1 MHz–2 GHz. The included averaging (avg = 8) of the VNA was used to reduce random errors.

**Figure 3:**
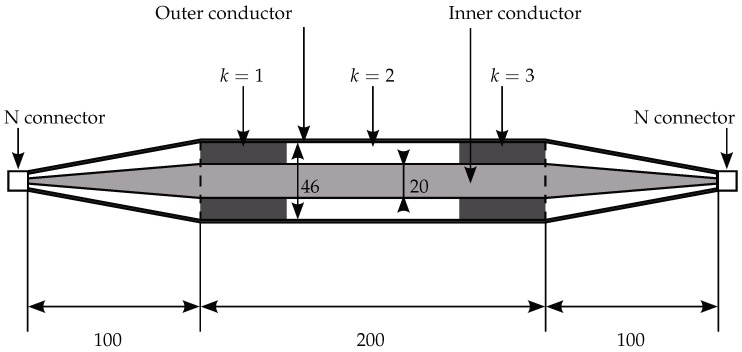
Coaxial cell [65] with the type N connector filled with PTFE (k=1 and k=3) and air (k=2); all dimensions in mm.

The measurements carried out in the coaxial cell [65] are shown in Figure 3. It was designed to only let the TEM mode propagate along the line below a cut-off frequency $f_{\mathrm{co}}=2.8\;\mathrm{GHz}$. The cell itself consists of three parts: the 200 mm sample holder section and the left and right transition sections from the smaller diameter of the type N connectors to the larger one of the sample holder. Furthermore, the geometry of the cell was designed to have a characteristic impedance of $Z_{0}=50\;\Omega$, and the phase velocity in air $c_{0}$ equaled the speed of light in a vacuum. The materials under test were 5 cm-thick cylinders made of PTFE, which can be inserted into the sample holder and perfectly fit the space between inner and outer conductors. Table 1 represents the layer setup, which was subsequently inverted.

All the measured scattering parameters refer to $Z_{0}$ impedance. Thus, the forward model had to refer to this value. This was done by adding zero thickness sections with $Z_{0}=50\;\Omega$ to the left and right end of the layered structure.

The $S_{ij}$-parameters measured by the VNA were calibrated and then used in the inversion algorithm (see Equations (10), (13)–(15)). For this case, the model parameters to be optimized were the frequency independent complex relative permittivity $\epsilon_{r,k}^{*}$ and the fractional position of layer intersection $d_{k}^{\prime }$. To avoid dependent parameters, fractional positions of $d_{k}^{\prime }$ of the fixed Device Under Test (DUT) thickness were used instead of the absolute layer thicknesses $d_{k}$. The losses in the PTFE layers were accounted for by the imaginary part of the permittivity $\epsilon_{r,k}^{\prime \prime }$ associated with every layer.

**Figure 4:**
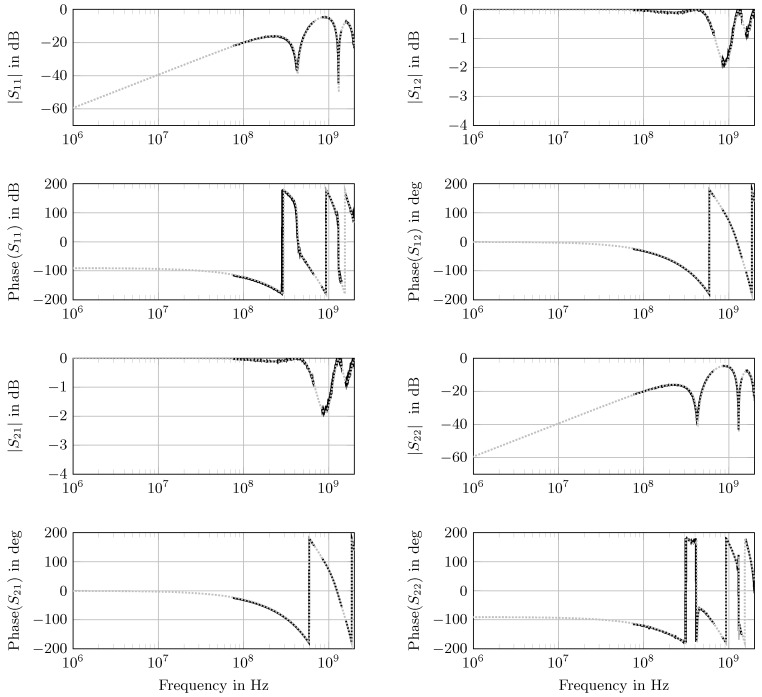
Scattering parameters $S_{ij}$ in magnitude and phase (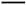) measured with the coaxial cell (three-layered setup: 50 mm PTFE, 100 mm air, 50 mm PTFE) and the corresponding fit (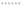).

Calibration was necessary to remove errors arising from the measurement equipment. The errors of the VNA and the cables until the end of the cable with the 3.5 mm connector were removed by a full two-port SOLT (Short, Open, Load, Thru) calibration, which is a standard method used with VNAs. Here, the electronic calibration module (N4431B, Agilent Technologies, Santa Clara, CA, USA) was used. The errors induced by the 3.5 mm to N adapters and the conical transition sections were phase shifts and losses (inductive, capacitive and ohmic). They were removed with three standards using a Thru Reflect Line calibration (TRL) based on [66]. The thru standard was realized by connecting the ports straight to each other. The reflect standard was carried out with an open port. The line standard was realized based on a matched transmission line with a known length. The scattering parameters of the three calibration measurements and the data from the DUT were the input for the algorithm from [66], which removes the influence of the transition sections. A disadvantages of the TRL calibration is the limited frequency band. This is because the phase shift in the transmission parameter induced by the line cannot be close to nπ with $n\in N_{0}^{+}$. Thus, the measured and calibrated frequency spectrum indicates gaps; see Figure 4. This can be solved by using different line lengths, which was presented in [67].

#### 2.4.2. Experiment 2: Synthetic Data with Dispersive Permittivity

**Figure A1:**
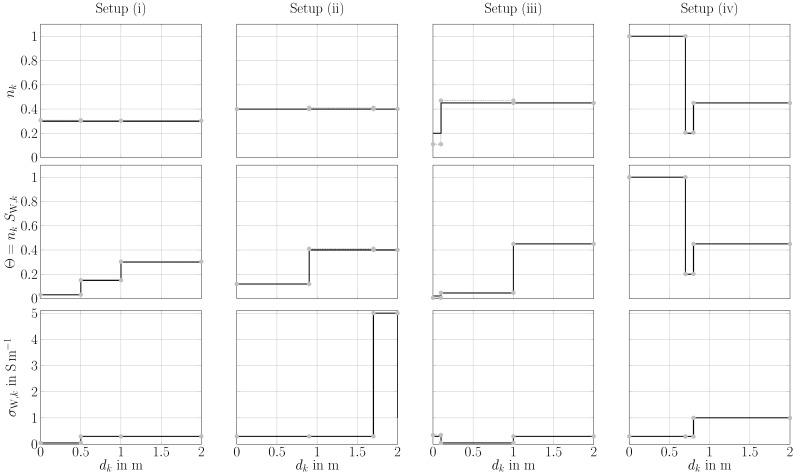
Comparison of the modeled (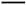) and retrieved layer properties (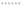) for all four setups.

To study the performance and precision of the algorithm in the case of dielectric dispersion and electrical losses, synthetic data corresponding to the setup presented in Table 2 were generated with the previously introduced forward model (see Equations (10), (13)–(15)). All four setups consisted of three layers with an overall thickness of 2 m and represented the following relevant hydrogeological setups (see Figure A1):

(i) Saturation of a dike body during a flood event from bottom or infiltration by rain from the top. In such a case, the water saturation $S_{W,k}$ is the key parameter because porosity is constant.
(ii) Salinization through groundwater intrusion in a coastal aquifer or usage of saline water in tracer tests. A high salt concentration leads to a high direct current electrical conductivity $\sigma_{W,k}$ and therefore influences electrical losses, as well as losses due to interactions between the pore solution and solid particles (e.g., Maxwell–Wagner effect).
(iii) The temporal hydraulic sealing of a top sandy soil layer by sedimentation of silt and clay fractions in the pore space. In such a case, porosity $n_{k}$ is reduced during the sedimentation, and the top layer of reduced $n_{k}$ becomes thicker.
(iv) The temporal hydraulic sealing of an aquifer under saturated conditions caused by sedimentation from silt and clay fractions during streaming of the pore space such as an infiltration or flooding. Even in saturated soil with its high EM attenuation, the temporal evolution of porosity can be estimated.

To model $\epsilon_{r,\mathrm{eff}}^{*}$ of each layer, the CRIM Formula (12) was used where the Debye parameters of water ($\epsilon_{\infty }$, $\epsilon_{s}$, τ) were fixed and taken from [68] at a temperature *T* = 20.6 °C. The permittivity of the solid particles used for all setups was assumed to be frequency independent and real with $\epsilon_{r,G}=5$, which is a justified approximation for organic free soils in the investigated frequency-temperature range [6,15].

Moreover, Gaussian noise was added to the modeled $\epsilon_{r,\mathrm{eff}}^{*}\left(\omega \right)$ of every layer. With a variance σ=0.1 of the normal distribution, a real measurement was simulated. The noise was applied to the real and imaginary part individually.

To analyze the possibilities and limitations of the inverse parameter estimation in a realistic remote sensing scenario without any prior information about the constitutive material properties of the porous material, only the dielectric relaxation of pure water and the matrix permittivity were assumed to be known. This was a justified assumption, due to the well-known dielectric properties of water if the temperature of the material were measured [25,47,69]. All parameters to be optimized are compiled in Table 2.

## 3. Results and Discussion

### 3.1. Coaxial Cell Results

In Figure 4, the forward model scattering parameters obtained from optimized material parameters are plotted together with the measurement taken with the coaxial cell.

In Table 3, the fitted parameters of the three layers are depicted. In the last column, the expected values for the materials used are given, which were taken from the producer’s datasheet. The fitted real parts $\epsilon_{r,k}^{\prime }$ and the thicknesses $d_{k}$ were in reasonable agreement with the expected values. Only the imaginary parts $\epsilon_{r,k}^{\prime \prime }$ differ. $\epsilon_{r,k}^{\prime \prime }$ refers to the losses of the material under test, which were relatively small for PTFE and air. The reason for the mismatch was that the sensitivity of the model for the scattering parameters of such low losses was too small, and hence, the value of the objective function did not change significantly when changing the parameter. This becomes clear when the variance was taken into account. It was approximately 10-times larger than the expected value, which means that the model and the measurement were not sensitive enough if the expected value were that small. However, the imaginary parts were all small and, thus, in the range of the expected values.

### 3.2. Synthetic Data Results

**Figure 5:**
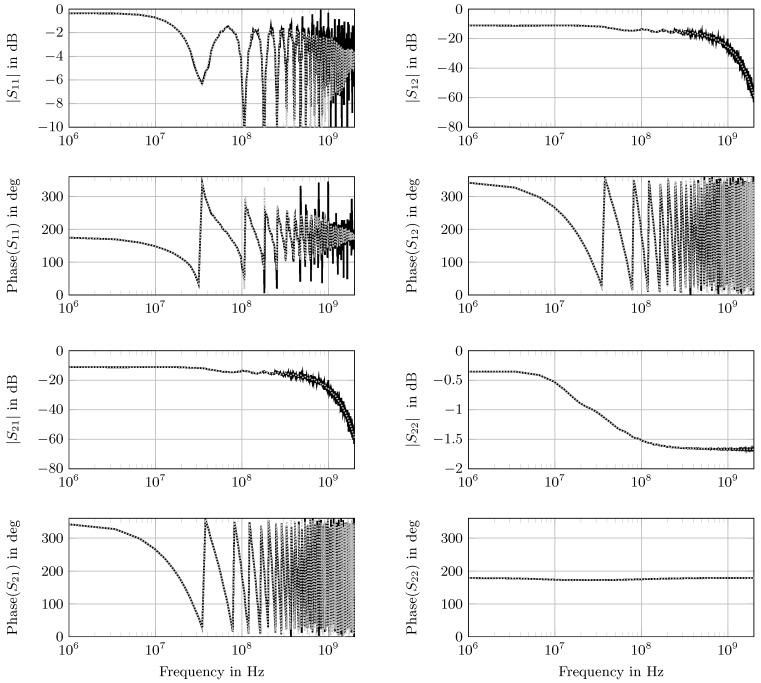
Synthetic scattering parameters $S_{ij}$ in magnitude and phase (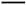) of the three-layered setup (iii) and the corresponding fit (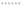). The resulting noise within the synthetic *S*-parameters together are added to the synthetically-generated spectral $\epsilon_{r,k}^{*}$-parameters on each layer.

Figure 5 represents the exemplary synthetically-generated *S*-parameters with parameters calculated from the fitted values for setup (iii). The selected setup (iii) is an example of a broad variation of different spectral properties of the individual layers (see Table 2) with a 0.1 m dry clay layer, a 0.9 m dry sand layer and the last 1 m sand layer saturated with tap water ($S_{W,3}=1$, $\sigma_{W,3}=0.3$ S m^−1^). The magnitude of $S_{11}$ demonstrates the typical graph of an unmatched line with the repeating resonances; while $S_{22}$ indicates the strong attenuation in the water saturated layer. Here, the incident wave was completely attenuated before reaching the transition to layer k=2. The magnitude of the reflection and the transmission was strongly reduced with increasing frequency, due to dispersion and absorption in the saturated layer. The four scattering parameters $S_{ij}$ calculated from the estimated parameter set were in reasonable agreement with the noisy synthetic data. At frequencies above 5 × 10^−8^ Hz, the absolute values of the predicted scattering parameters showed a systematic deviation. This was due to two reasons: (i) it was an apparent effect due to the linear sampling; and (ii) the equally applied noise of $\epsilon_{r,\mathrm{eff}}^{*}$ was multiplied with the frequency in the scattering parameter calculation (see Equations (1) and (3) in Section 2.1). Hence, the influence of the noise enhanced with rising frequency.

**Figure 6:**
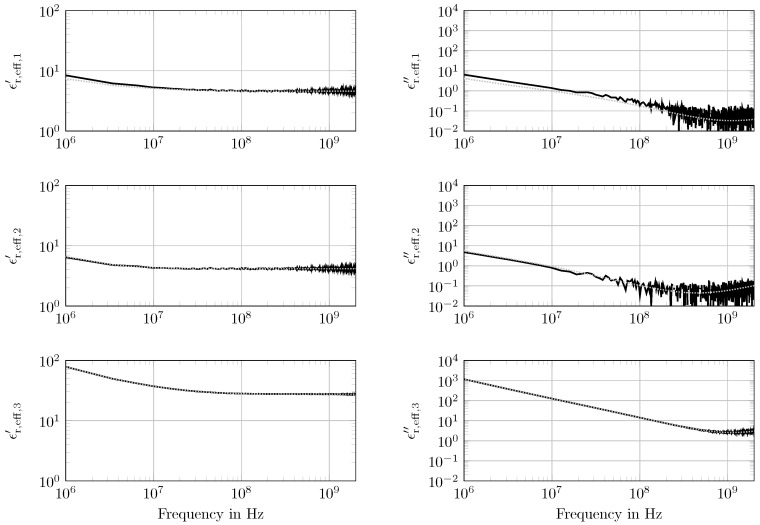
Spatial retrieval of the broadband dielectric spectra for setup (iii). Synthetic spectra (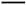) and the retrieved broadband dielectric spectra (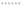).

In Figure 6, the forward modeled broadband spectra including the noise are plotted together with the retrieved spectra obtained from inverted material parameters. For the three layers, the spectrum is plotted separately. If $\epsilon_{r,\mathrm{eff},k}^{\prime \prime }$ were high, the noise had less influence, as can be seen in the plots. Because of the high $\epsilon_{r,\mathrm{eff},3}^{\prime \prime }\left(f\right)$ values, respectively water content and the thickness of $d_{3}=1\;m$, the parameter of layer k=3 had a strong influence on the objective function. This means that the parameter had been estimated close to the model parameter, and the reconstructed spectra matched well with the noisy ones. However, for the thinner layers with low water content, the spectra indicated a deviation (see Table A1 and Figure A1). The mismatch of the spectra of layer k=1 was stronger than that of layer k=2. The reason here was the lower influence of the layer parameters on the overall result. Especially the thin layer k=1 ($d_{1}=0.1\;m$) had a low influence, and therefore, the parameters had not been calculated precisely. However, also the water content played an important role: the water content mainly determined the losses, which furthermore caused the reduction of the transmission magnitude at higher frequencies. However, there was a considerable deviation in the real parts of layer k=1 and k=2 at high frequencies (f=2 GHz: $\epsilon_{r,\mathrm{eff},1}^{\prime }\approx 4.8$ and $\epsilon_{r,\mathrm{eff},2}^{\prime }\approx 4$). The reason for that was the lower porosity of layer k=1, and consequently, it had a higher percentage of solid material. This showed that the information about, e.g., the porosity was present in the permittivity spectra. Generally, the presented method enabled the retrieval of dielectric relaxation spectra.

**Figure 7:**
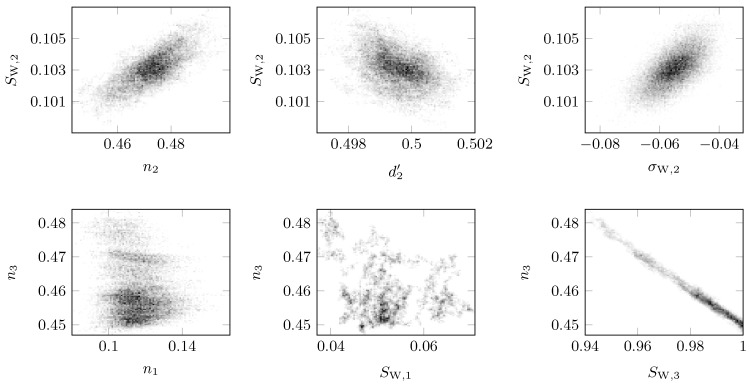
Selected correlation plots for setup (iii). Only some significant and typical plots, like strong correlation ($n_{3}$ vs. $S_{W,3}$), no correlation ($S_{W,2}$ vs. $d_{2}^{\prime }$), weak correlation ($S_{W,2}$ vs. $\sigma_{W,2}$) etc., are shown. The other correlation plots were similar to the displayed ones.

For layers k=2 and k=3, the fitted parameters (see Table A1) were in reasonable agreement with the model parameters, and the uncertainty ranges were lower, 1% of the fitted values. Only the one of the direct current conductivity $\sigma_{W,2}$ of layer k=2 was about 13%. However, the water content and the conductivity of this layer were relatively low and resulted in low imaginary parts $\epsilon_{r,\mathrm{eff},2}^{\prime \prime }\left(f\right)$. Because of that, the noise became more crucial at high frequencies, and so, it was difficult to estimate the parameters during the optimization. However, even if the uncertainty range was added to and subtracted from the fitted value, the conductivity stayed in the typical dry loamy sand range and was clearly distinguished from the higher conductivity of layer k=3. The fitted parameters of layer k=1 did not match that precisely. Only the value of the thickness $d_{1}^{\prime }$ was in good agreement with the model parameter. The other values led to a considerable mismatch. Especially the saturation $S_{W,1}$ had a difference of one order of magnitude. On the one hand the parameters of such thin layers with low losses and low contrast to the adjacent layers had a low influence on the objective function and thus had not been estimated precisely. On the other hand, there were correlations between the parameters. If, e.g., the porosity and the saturation were underestimated, the conductivity had to increase to get the same layer loss. This was described in detail together with the correlation plots in Figure 7. However, the relevant parameter of this experiment was the porosity, which was underestimated, but still reasonably lower than the one of layer k=2. Therefore, the contrast of porosity between the two layers was clearly visible, and the clogging of a sand layer with former high hydraulic conductivity by sedimentation was detected here.

The mentioned correlations caused problems in the optimization and especially in the MCMC uncertainty estimation. If the Gelman–Rubin criterion in Equation (16) was not met after several thousands of model iterations for all parameters, it was unlikely that it would be after more iterations. The reason for that was the dependency of the parameters. Here, the correct uncertainty range was not determined. If more iterations would have been carried out, the range would have only become slightly smaller. The correct range then would have to be smaller than the current one, as well. However, the iterations were stopped if almost all ranges were under 10% of the fitted value. Uncertainties that did not converge were marked with a “No” in the convergence column (Con.) in Table A1.

Mainly the ranges for porosity and saturation did not converge, because the parameters were correlated. This correlation can be explained as follows: If the overall loss sum of the whole setup was fixed, the volumetric water content was fixed, as well, especially at high conductivities. This was because the losses strongly depended on the losses in the water. However, if the water content was fixed and the saturation was underestimated, the porosity had to increase. This can be seen clearly in the correlation plot of $S_{W,3}$ and $n_{3}$ in Figure 7. The expected relationship between the two parameters was evident. Every parameter pair in the plot led to the same optimum, and the given uncertainty range was the width of the data cloud. If parameters were correlated, the cloud was stretched, and the range was taken from the end points and consequently tended to be relatively wide. No reasonable conclusion about the quality of the optimum could be drawn here. In principle, every point in the cloud could be the optimum with the same probability. Especially for high water contents, this correlation was relevant.

The plot of $d_{2}^{\prime }$ versus $S_{W,2}$ indicates an expected uncorrelated shape. The sampled data points were distributed over a filled circle around the estimated optimal parameter. This means that this parameter set converged to a normal distribution. In this case, the mean was the most likely parameter, and the given uncertainty range converged fast enough and equaled the variance of the normal distribution. The thickness fraction $d_{2}^{\prime }$ resulted in a low correlation to other parameters. Theoretically there was a slight correlation to the permittivity $\epsilon_{r,\mathrm{eff},2}^{\prime }$, because both determined the position of the resonances in the reflection parameter $S_{11}$. However, if the water content was low, $\epsilon_{r,\mathrm{eff},2}^{\prime }$ was mainly determined by the solid material permittivity $\epsilon_{r,G}^{\prime }$, which was fixed in this experiment. Thus, only the thickness affected the position of the resonances, which in turn highly influenced the objective function. Consequently, the thickness was set easily by the optimization algorithm.

The plots $\sigma_{W,2}$ over $S_{W,2}$ and $n_{2}$ over $S_{W,2}$ showed a similar shape as the former one. However, the cloud was a little slimmer and indicated a slight correlation. Because of the weak correlation the uncertainty range estimation converged fast enough, but no normal distribution of the samples around the optimum was present. However, the uncertainty ranges of the parameters were plausible. Even from weak correlations, information about the model was gained. The correlation between saturation and porosity were already explained. Because of the low water content in layer k=2, it was weaker than in layer k=3. The relationship between the direct current conductivity $\sigma_{W,2}$ and the saturation $S_{W,2}$ was again explained with the help of the losses. The conductivity of the water strongly influenced the losses. However, if the overall losses were fixed and the water content rose with the saturation, conductivity had to decrease to result in the same overall loss.

The plot of $n_{1}$ over $n_{3}$ was a typical plot for an uncorrelated parameter pair, where the uncertainty range calculation did not converge fast enough. Perhaps there was a complex relationship that was not visible in the correlation plot. The given uncertainty range was only evaluated qualitatively and should have been under 10% of the fitted parameter. In any case, if the range were added or subtracted, one should have been able to distinguish between different results of the experiment. Otherwise, the parameter set was not useful to make reasonable statements. These two parameters met this requirement.

Similarly good results were achieved with the other setups (see Table A1 and Figure A1). All fitted values matched with the model parameters, and almost all uncertainty ranges were close to or under 10% of the fitted value. Only the ranges of setup (i) $\sigma_{W,1}$ and setup (iii) $S_{W,1}$ were problematic. Setup (iii) was discussed in detail before. The uncertainty range of $\sigma_{W,1}$ in setup (i) was in the same order of magnitude as the fitted value. However, the water content and the direct current conductivity were relatively small and thus had a low influence on the overall loss. This was not a problem because this experiment focused on water content. As was described in setup (iii), the correlation between $n_{3}$ and $S_{W,3}$ caused problems in the MCMC uncertainty calculation if the saturation and thus the water content were high. However, good results had been achieved with the Gelman–Rubin criterion (16).

If the different focuses of the four setups were taken into account, all experiments were successful. Setup (i) was a typical water content experiment: three differently saturated layers with the same porosity were detected. This means the different water contents were found correctly by the algorithm. Setup (ii) was a salination experiment. The key parameter here was the direct current conductivity $\sigma_{W,k}$. The value of layer k=3 was almost one order of magnitude higher than the one of layer k=2. Thus, the conductivity increase in the bottom layer was detected. Setup (iii) was already explained together with die spectra plots. Setup (iv) was again a porosity experiment, but here under saturated conditions. Here, the advantage of measuring the $S_{ij}$-parameter from two sides was obvious. In setup (iv), the measurement from above could not be used since the losses in the water layer k=1 were too high. Nevertheless, the underlying layers k=2 and k=3 were well reconstructed, and the porosity difference from layer k=2 to k=3 was well detected.

In general, it should be noted that the retrieval worked well for high porosities and high dispersive properties due to total saturation. The mixed material dispersion and absorption spectra were calculated with the reconstructed parameters. Hence, the proposed method allowed the reconstruction of complex layer setups and enabled the estimation of spatially-distributed spectral parameters of dispersive and electrically-lossy porous media.

## 4. Conclusions

In this article, a high frequency broadband EM retrieval approach (1 MHz–2 GHz) for a layered half space was suggested. The core of the retrieval scheme used was a frequency domain two-port transmission line forward model according to [52] including a frequently-used power law soil mixture model (Complex Refractive Index Model (CRIM), [28]) considering (i) the volume fractions of the soil phases; (ii) dielectric relaxation of the aqueous pore solution; (iii) electrical losses and (iv) low frequency dispersion due to the interactions between the pore solution and solid particles. This enabled the realistic consideration of dispersion, as well as electrical and dielectric losses and thus the improved determination of spatial distributed dielectric relaxation behavior of the soil in comparison to available approaches by [29,30,31,32,33,70]. However, the mixture approach used was a three-phase model of the soil, and the implied frequency characteristics of the expected dielectric spectra indicated the typical signature due to the model structure shown by [25].

The spatially-distributed retrieval of broadband dielectric spectra was achieved with a global optimization approach based on a Shuffled Complex Evolution (SCE) algorithm in, e.g., four scenarios: (i) water saturation of a dike in a flood event from the bottom or infiltration by rain from the top; (ii) salinization of groundwater intrusion in a coastal aquifer; (iii) hydraulic sealing of the top sandy soil by sedimentation or (iv) an aquifer under saturated conditions. Estimated parameters in the broadband mixture model were thickness, porosity, water saturation and electrical conductivity of the aqueous pore solution of each soil layer. The possibilities and limitations of the inverse parameter estimation in these four scenarios of practical relevance were numerically analyzed without any prior information about the material properties of the porous material.

The reasonable agreement of expected and optimized soil properties in each scenario showed that the developed model was suitable for a retrieval kernel. In every layer, only four soil parameters were adjustable by the inversion algorithm. This makes it possible to estimate several layers quickly at the same time and thus reconstruct in-homogeneous material parameter distributions. Moreover, the proposed frequency domain transmission line approach allows an automatic adaptation of layer number and thickness in contrast to previous schemes, which were used with regular grids in time and/or space [30,31,34,70].

Improvement of this kind of investigation can be realized by applying a noise model based on adding an uncertainty to the model parameters instead of the effective relative dielectric permittivity. Moreover, an adaption of the CRIM formula can be helpful: the porosity can be fixed using prior knowledge, and then, the saturation can be replaced by the volumetric water content, or the permittivity of the solid particles can be fixed.

## Figures and Tables

**Table 1:** Experimental setup for coaxial cell measurement.

Layer	Material	$d_{k}$ Thickness	$d_{k}^{\prime }$ Fraction
k=1	PTFE	50 mm	0.25
k=2	Air	100 mm	0.75
k=3	PTFE	50 mm	1

**Table 2:** Experimental setups of the synthetic data.

Setup	Layer	Description	$d_{k}$	$d_{k}^{\prime }$	$n_{k}$	$S_{W,k}$	$\sigma_{W,k}$
(i)	k=1	Dry	0.5 m	0.25	0.3	0.1	0.05 S m^−1^
	k=2	Partly saturated with tap water	0.5 m	0.5	0.3	0.5	0.3 S m^−1^
	k=3	Saturated with tap water	1 m	1	0.3	1	0.3 S m^−1^
(ii)	k=1	Partly saturated with tap water	0.9 m	0.45	0.4	0.3	0.3 S m^−1^
	k=2	Saturated with tap water	0.8 m	0.85	0.4	0.1	0.3 S m^−1^
	k=3	Saturated with sea water	0.3 m	1	0.4	1	1 S m^−1^
(iii)	k=1	Dry clay	0.1 m	0.05	0.2	0.1	0.3 S m^−1^
	k=2	Dry sand	0.9 m	0.5	0.45	0.1	0.05 S m^−1^
	k=3	Saturated sand with tap water	1m	1	0.45	1	0.3 S m^−1^
(iv)	k=1	Stream water	0.7 m	0.35	1	1	0.3 S m^−1^
	k=2	Saturated sand with sedimentation	0.1 m	0.4	0.2	1	0.3 S m^−1^
	k=3	Saturated sand with water	1.2 m	1	0.45	1	0.1 S m^−1^

**Table 3:** Results of the coaxial cell reconstruction (expected values for PTFE are taken from the producer datasheet).

Layer	Parameter	Fit	Uncertainty Range	Expected
1	$\epsilon_{r,1}^{\prime }$	2.00	1.57 × 10^−3^	2
	$\epsilon_{r,1}^{\prime \prime }$	9.69 × 10^−8^	1.51 × 10^−3^	4 × 10^−4^
	$d_{1}^{\prime }$	0.25	0.16 × 10^−3^	0.25
	$d_{1}$ in mm	50	3.2 × 10^−2^	50
2	$\epsilon_{r,2}^{\prime }$	1.01	0.54 × 10^−3^	1
	$\epsilon_{r,2}^{\prime \prime }$	1.99 × 10^−3^	0.53 × 10^−3^	0
	$d_{2}^{\prime }$	0.75	0.16 × 10^−3^	0.75
	$d_{2}$ in mm	100	3.2 × 10^−2^	100
3	$\epsilon_{r,3}^{\prime }$	2.04	1.64 × 10^−3^	2
	$\epsilon_{r,3}^{\prime \prime }$	9.71 × 10^−7^	1.50 × 10^−3^	4 × 10^−4^
	$d_{3}^{\prime }$	Fixed	-	1
	$d_{3}$ in mm	Fixed	-	50

## Abbreviations

Con	Convergence
CRIM	Complex Refractive Index Model
DUT	Device Under Test
EM	Electromagnetic
FD	Frequency Domain
GPR	Ground Penetrating Radar
GR	Gelman–Rubin criterion
HF	High Frequency
MCMC	Monte Carlo Markov Chain
PTFE	Polytetrafluorethylene
SCE	Shuffled Complex Evolution
SOLT	Short, Open, Load, Thru
TD	Time Domain
TDR	Time Domain Reflectometry
TEM	Transverse Electromagnetic
TRL	Thru, Reflect, Line
Uncert	Uncertainty Range
VNA	Vector Network Analyzer

## Appendix A

**Table A1:** Optimized parameters of synthetic data (Con. = Convergence, Uncert. = Uncertainty Range).

		Setup (i)				Setup (ii)				Setup (iii)				Setup (iv)			
Layer	Parameter	Model	Fit	Uncert.	Con.	Model	Fit	Uncert.	Con.	Model	Fit	Uncert.	Con.	Model	Fit	Uncert.	Con.
k=1	$n_{1}$	3.0 × 10${}^{-1}$	3.08 × 10${}^{-1}$	7.6 × 10${}^{-3}$		4 × 10${}^{-1}$	4 × 10${}^{-1}$	2.7 × 10${}^{-3}$		2 × 10${}^{-1}$	1.1 × 10${}^{-1}$	1.12 × 10${}^{-2}$	No	1	1	6.57 × 10${}^{-5}$	
	$S_{W,1}$	1 × 10${}^{-1}$	1.01 × 10${}^{-1}$	1.1 × 10${}^{-3}$		3 × 10${}^{-1}$	3 × 10${}^{-1}$	9.2 × 10${}^{-4}$		1 × 10${}^{-1}$	4.8 × 10${}^{-2}$	2.84 × 10${}^{-2}$	No	1	1	5.46 × 10${}^{-5}$	
	$\sigma_{W,1}$ in S m${}^{-1}$	5 × 10${}^{-2}$	4.89 × 10${}^{-2}$	4.62 × 10${}^{-2}$		3 × 10${}^{-1}$	3 × 10${}^{-1}$	2 × 10${}^{-3}$		3 × 10${}^{-1}$	3.5 × 10${}^{-1}$	3.95 × 10${}^{-6}$		3 × 10${}^{-1}$	3 × 10${}^{-1}$	7.08 × 10${}^{-5}$	
	$d_{1}^{\prime }$	2.5 × 10${}^{-1}$	2.5 × 10${}^{-1}$	3.56 × 10${}^{-4}$		4.5 × 10${}^{-1}$	4.5 × 10${}^{-1}$	1.9 × 10${}^{-4}$		5 × 10${}^{-2}$	4.89 × 10${}^{-2}$	1.9 × 10${}^{-4}$		3.5 × 10${}^{-1}$	3.5 × 10${}^{-1}$	3.61 × 10${}^{-5}$	
	$d_{1}$ in m	5 × 10${}^{-1}$	5 × 10${}^{-1}$	1.12 × 10${}^{-4}$		9 × 10${}^{-1}$	9 × 10${}^{-1}$	3.8 × 10${}^{-4}$		1 × 10${}^{-1}$	1 × 10${}^{-1}$	3.8 × 10${}^{-4}$		7 × 10${}^{-1}$	7 × 10${}^{-1}$	7.22 × 10${}^{-4}$	
k=2	$n_{2}$	3 × 10${}^{-1}$	3.03 × 10${}^{-1}$	1.6 × 10${}^{-3}$	No	4 × 10${}^{-1}$	4 × 10${}^{-1}$	5.4 × 10${}^{-2}$	No	4.5 × 10${}^{-1}$	4.7 × 10${}^{-1}$	1 × 10${}^{-2}$		2 × 10${}^{-1}$	2.06 × 10${}^{-1}$	7.8 × 10${}^{-3}$	No
	$S_{W,2}$	5 × 10${}^{-1}$	4.97 × 10${}^{-1}$	1.63 × 10${}^{-2}$	No	1	1	1 × 10${}^{-1}$	No	1 × 10${}^{-1}$	1 × 10${}^{-1}$	1.3 × 10${}^{-3}$		1	9.83 × 10${}^{-1}$	5.37 × 10${}^{-2}$	No
	$\sigma_{W,2}$ in S m${}^{-1}$	3 × 10${}^{-1}$	2.96 × 10${}^{-1}$	7.8 × 10${}^{-3}$	No	3 × 10${}^{-1}$	3 × 10${}^{-1}$	7.5 × 10${}^{-3}$	No	5 × 10${}^{-2}$	5.4 × 10${}^{-2}$	9.6 × 10${}^{-3}$		3 × 10${}^{-1}$	2.9 × 10${}^{-1}$	9.6 × 10${}^{-3}$	
	$d_{2}^{\prime }$	5 × 10${}^{-1}$	5 × 10${}^{-1}$	1.9 × 10${}^{-4}$		8.5 × 10${}^{-1}$	8.5 × 10${}^{-1}$	8.4 × 10${}^{-4}$		5 × 10${}^{-1}$	5 × 10${}^{-1}$	6.61 × 10${}^{-4}$		4 × 10${}^{-1}$	4 × 10${}^{-1}$	4 × 10${}^{-3}$	
	$d_{2}$ in m	5 × 10${}^{-1}$	5 × 10${}^{-1}$	3.8 × 10${}^{-4}$		8 × 10${}^{-1}$	8 × 10${}^{-1}$	3.8 × 10${}^{-4}$		9 × 10${}^{-1}$	9 × 10${}^{-1}$	1.3 × 10${}^{-3}$		1 × 10${}^{-1}$	1 × 10${}^{-1}$	8 × 10${}^{-3}$	
k=3	$n_{3}$	3 × 10${}^{-1}$	3.04 × 10${}^{-1}$	3.8 × 10${}^{-3}$	No	4 × 10${}^{-1}$	4 × 10${}^{-1}$	6 × 10${}^{-3}$	No	4.5 × 10${}^{-1}$	4.5 × 10${}^{-1}$	1.81 × 10${}^{-2}$	No	4.5 × 10${}^{-1}$	4.5 × 10${}^{-1}$	5.26 × 10${}^{-4}$	No
	$S_{W,3}$	1	1	1.6 × 10${}^{-2}$	No	1	1	1.2 × 10${}^{-2}$	No	1	1	4.11 × 10${}^{-2}$	No	1	1	1.4 × 10${}^{-3}$	No
	$\sigma_{W,3}$ in S m${}^{-1}$	3 × 10${}^{-1}$	3 × 10${}^{-1}$	1.6 × 10${}^{-3}$		5	5	1.2 × 10${}^{-2}$	No	3 × 10${}^{-1}$	3 × 10${}^{-1}$	2.5 × 10${}^{-3}$	No	1 × 10${}^{-1}$	1 × 10${}^{-1}$	5.66 × 10${}^{-5}$	
	$d_{3}^{\prime }$	1	Fixed	-		1	Fixed	-		1	Fixed	-		1	Fixed	-	
	$d_{3}$ in m	1	Fixed	-		3 × 10${}^{-1}$	Fixed	-		1	Fixed	-		1.2	Fixed	-	
